# Aberrant Functional Connectivity of the Orbitofrontal Cortex Is Associated With Excited Symptoms in First-Episode Drug-Naïve Patients With Schizophrenia

**DOI:** 10.3389/fpsyt.2022.922272

**Published:** 2022-07-28

**Authors:** Congxin Chen, Jingjing Yao, Yiding Lv, Xiaoxin Zhao, Xinyue Zhang, Jiaxi Lei, Yuan Li, Yuxiu Sui

**Affiliations:** ^1^Department of Psychiatry, Affiliated Nanjing Brain Hospital, Nanjing Medical University, Nanjing, China; ^2^Jinhu People’s Hospital, Jinhua, China; ^3^Shanghai Mental Health Center, Shanghai Jiao Tong University School of Medicine, Shanghai, China; ^4^Shenzhen KangNing Hospital, Shenzhen, China; ^5^Chengdu No. 4 People’s Hospital, Chengdu, China

**Keywords:** schizophrenia, orbitofrontal cortex, fractional amplitude of low-frequency fluctuations, functional connectivity, hostility, impulsivity

## Abstract

**Background:**

Schizophrenia (SZ) is associated with the highest disability rate among serious mental disorders. Excited symptoms are the core symptoms of SZ, which appear in the early stage, followed by other stages of the disease subsequently. These symptoms are destructive and more prone to violent attacks, posing a serious economic burden to the society. Abnormal spontaneous activity in the orbitofrontal cortex had been reported to be associated with excited symptoms in patients with SZ. However, whether the abnormality appears in first-episode drug-naïve patients with SZ has still remained elusive.

**Methods:**

A total of 56 first-episode drug-naïve patients with SZ and 27 healthy controls underwent resting-state functional magnetic resonance imaging (rs-fMRI) and positive and negative syndrome scale (PANSS). First, differences in fractional amplitude of low-frequency fluctuations (fALFF) between first-episode drug-naïve patients with SZ and healthy controls were examined to identify cerebral regions exhibiting abnormal local spontaneous activity. Based on the fALFF results, the resting-state functional connectivity analysis was performed to determine changes in cerebral regions exhibiting abnormal local spontaneous activity. Finally, the correlation between abnormal functional connectivity and exciting symptoms was analyzed.

**Results:**

Compared with the healthy controls, first-episode drug-naïve patients with SZ showed a significant decrease in intrinsic activity in the bilateral precentral gyrus, bilateral postcentral gyrus, and the left orbitofrontal cortex. In addition, first-episode drug-naïve patients with SZ had significantly reduced functional connectivity values between the left orbitofrontal cortex and several cerebral regions, which were mainly distributed in the bilateral postcentral gyrus, the right middle frontal gyrus, bilateral paracentral lobules, the left precentral gyrus, and the right median cingulate. Further analyses showed that the functional connectivity between the left orbitofrontal cortex and the left postcentral gyrus, as well as bilateral paracentral lobules, was negatively correlated with excited symptoms in first-episode drug-naïve patients with SZ.

**Conclusion:**

Our results indicated the important role of the left orbitofrontal cortex in first-episode drug-naïve patients with SZ and suggested that the abnormal spontaneous activity of the orbitofrontal cortex may be valuable to predict the occurrence of excited symptoms. These results may provide a new direction to explore the excited symptoms of SZ.

## Introduction

Schizophrenia (SZ) is one of the most serious and disabling mental diseases (1). However, due to the complexity of brain structure and functional activities, the etiology of SZ has not been fully clarified yet. Researchers have put forward a series of hypotheses, demonstrating that abnormal cerebral blood flow and neurobiochemical activities may lead to clinical symptoms (2–6). A large number of studies have pointed out that compared with healthy individuals, SZ patients are at a significantly increased risk of excited symptoms (including excitement, hostility, uncooperativeness, and impulsivity) by 2.1–4.6 times (7, 8). The sudden events, such as destroyed items and violent assaults, are mainly associated with excited symptoms in patients with SZ, which have posed a great burden to society (9–13). Patients with excited symptoms may experience substance abuse, which may prolong the treatment cycle and deteriorate clinical treatment results (13–15). Nevertheless, few clinical studies have yet concentrated on excited symptoms in patients with SZ.

Hostility is the expression of anger or resentment toward others in terms of words or actions (13). Impulsivity is an obstacle to the regulation and control of internal impulse response when integrating internal and external stimuli (16–19), which may lead to reckless emotional catharsis and violence (20, 21). Patients with SZ lack the ability to timely deal with negative emotions. That is to say, they are highly sensitive to negative emotions and prone to negative urgency (10, 21). In SZ patients with delusions and hallucinations, sudden impulsive behavior in the face of stress or other extreme events is caused by uncontrolled internal regulation of emotional responses, as well as imbalance in external motor inhibition (16, 22). Such impulsive behaviors, which not accompany by poor prognostic outcomes, but also increase the burden of disease, are highly prevalent in patients with first-episode and chronic SZ (22–24). Hostility and impulsivity are the most problematic symptoms in patients with SZ (13). The study of impulsivity and hostility in patients with excited symptoms may provide new ideas for understanding the neuropathological mechanisms of SZ (25).

Hostility and impulsivity are closely associated with dysfunction of the prefrontal cortex, especially the orbitofrontal cortex (OFC) (26). Studies have shown that impulsive, hostile, and antisocial behaviors are perpetrated by healthy individuals after OFC damage (27). OFC serves as a central region of emotional integration and inhibitory control (28). Alterations in this cerebral region have been demonstrated to be strongly associated with impulsive behaviors in patients with psychiatric disorders (18, 26). Structural magnetic resonance imaging (MRI) studies suggested that patients with SZ who were more impulsive showed larger gray and white matter volumes, as well as a smaller cortical thickness in the OFC area compared with those with no history of impulsivity (10, 29). Functional MRI studies revealed that patients with impulsivity showed anomalous functional connections between the OFC and anterior cingulate gyrus (19, 30). In addition, several MRI studies demonstrated that the reduced gray matter volume in OFC is negatively correlated with the severity of emotional instability, and an abnormal OFC function has also been shown to be associated with the expressions of negative emotions, such as anxiety and fear in patients with SZ (4, 6, 31, 32). OFC regulates negative emotions by receiving and transmitting relevant emotional information. After functional impairment, it shows emotional vulnerability and irritability (3, 5, 32, 33). Given that the OFC is a central element in the emotional and behavioral response network and plays a central role in both emotional regulation and motor inhibition (28), it is hypothesized that the dysfunction of the OFC is the basis of the manifestation of excited symptoms in patients with SZ.

OFC neurons are common targets for classic and glutamatergic antipsychotic drugs (34). After drug therapy for patients with chronic SZ, the functional activity of OFC increases significantly, and its association with excited symptoms may be affected (35). Previous studies could not well exclude the above-mentioned confounding factors (36). Therefore, after removing the effects of the course of the disease, the long-term use of antipsychotics, and progressive cerebral atrophy on OFC function, exploring the damage to OFC function in patients with first-episode SZ may assist clinicians in the early identification or remission of excited symptoms.

Resting-state functional magnetic resonance imaging (rs-fMRI) is emerging as a valuable biomarker for measuring connectivity of the brain in patients with neural disorders. It can non-invasively measure internal neural activity with a great stability. The amplitude of low-frequency fluctuations (ALFF) is the most commonly used index to measure the intensity of regional spontaneous cerebral activities (37, 38). However, when capturing the relative amplitude of blood oxygen level-dependent (BOLD) signal fluctuation in gray matter, a higher frequency range (> 0.08 Hz) of ALFF is sensitive to the interference of physiological factors, such as heartbeat, respiration, etc. (31, 37, 39). The fractional ALFF (fALFF) method provides a relative estimate of the BOLD signal intensity in the relevant frequency band (0.01–0.08 Hz) (37, 40). After significantly lessening non-specific signal components, the fALFF improved the sensitivity and specificity in detecting spontaneous cerebral activities (37, 41). As another frequently used index, resting-state functional connectivity (rs-FC) analysis describes the functional correlation among spatially unconnected cerebral regions (42, 43). Preliminary studies of SZ have reported abnormal fALFF in complex cerebral regions. The fALFF-based resting-state functional connectivity (rsFC) analytical strategy has been scarcely utilized to examine the functional interactions of cerebral regions that showed an altered fALFF in drug-naïve patients with SZ.

The present cross-sectional study has mainly concentrated on OFC to explore abnormalities in the spontaneous cerebral activity of first-episode drug-naïve patients with SZ. A great number of patients with the first onset of SZ who received few medical treatments, as well as age-, gender-, educational level-matched healthy controls (HCs) were recruited. All subjects underwent fMRI scan and positive and negative syndrome scale (PANSS). Firstly, we attempted to determine the abnormal spontaneous cerebral activity in first-episode drug-naïve patients with SZ, which was measured by fALFF. It was supposed that the neural networks associated with OFC would be vulnerable. Secondly, we considered cerebral regions found in the previous fALFF analysis to compare the functional cerebral activities between first-episode drug-naïve patients with SZ and HCs. Finally, to indicate whether the abnormalities found in the fALFF and fALFF-based rsFC analyses would be associated with excited symptoms in patients with SZ, we analyzed the correlation between abnormal indices and scores and sub-scores of excited symptoms.

## Materials and Methods

The Ethics Committee of the Nanjing Brain Hospital (Nanjing, China; Approval No. 2013KY010) approved the study protocol. All participants signed the written informed consent form prior to enrollment.

### Subjects

We used the previously reported data (36, 44). First-episode drug-naïve patients with SZ (*n* = 63, 44 men) were recruited from Nanjing Brain Hospital. Patients were diagnosed *via* Structured Clinical Interview for Diagnostic and Statistical Manual of Mental Disorders, fourth edition (DSM-IV) (SCID-I/P), and met the criteria for SZ. In total, 30 HCs (17 men and 13 women) who were matched in age, educational level, and gender were recruited through advertisements in the local community of Nanjing city. An experienced psychiatrist assessed the current state of mind and personal or family history of any mental disorders. None of the HCs presented a personal or family history of psychiatric disorders.

The inclusion criteria were as follows: (1) patients who aged 15–45 years old, (2) right-handed patients, and (3) an ability to understand the instructions and contents of the survey. The exclusion criteria were as follows: (1) a history of head injury, seizures, cerebrovascular disease, neurological diseases (excluding SZ), impaired thyroid function, (2) disability in learning, and (3) meeting DSM-IV criteria for alcohol or substance abuse or dependence in the past year.

### Psychological Assessment of Patients

Two trained specialists who had no idea of the content of the research assessed the mental state of patients using the PANSS on the day of the fMRI test. The PANSS originally designed by Kay et al. was used to better evaluate the heterogeneity of symptoms in patients with SZ (45–47). Positive factors, such as delusions, hallucinations, and unusual thought content are mainly considered by clinicians for inclusion of patients. Negative factors (e.g., motivation and social withdrawal, blunted affect, as well as motor retardation) were reported as prognostic factors (32). General psychopathology is composed of excited factors (mainly involving impulsive behaviors), disorganized/concrete factors (conceptual disorganization, difficulty in abstract thinking, stereotyped thinking, and poor attention), and depressed factors (anxiety, tension, guilt feelings, and depression) (46). The total score and five-factor scores of PANSS were assessed with SCID-I/P to measure the severity of psychotic symptoms.

### Imaging Acquisition

MRI was performed within 3 days after confirmation of the diagnosis of SZ. The MRI data were collected using the 3.0-T Siemens Verio scanner (Siemens Healthcare, Erlangen, Germany). Besides, foam pads and soft earplugs were used to reduce head motion and scanning noise. Sagittal three-dimensional T1-weighted images were obtained using cerebral volume sequences with the following parameters: repetition time (TR) = 2,000 ms; echo time (TE) = 2.3 ms; reversal time = 900 ms; flip angle (FA) = 7°; field of view (FOV) = 256 mm × 256 mm; matrix size = 256 × 256; slice thickness = 1 mm, gap = 0.5 mm; sagittal section; and acquisition time = 353 s. The gradient echo single-shot echo-planar imaging sequence was used to collect functional BOLD images in the resting-state. The following parameters were considered: TR/TE = 2,000/30 ms; FOV = 240 mm × 240 mm; matrix size = 64 × 64; FA = 90°; slice thickness = 4 mm; no gap; 30 transverse cross-sections; 171 volumes; acquisition time = 346 s. All subjects were asked to lie quietly with their eyes closed, relax, and move as little as possible, without thinking about anything, especially avoiding fall asleep during data collection.

### Data Preprocessing

There were significant differences in the original data, which could not be analyzed directly, and the fMRI data were preprocessed using the MATLAB 2014a software and Data Processing Assistant for rs-fMRI (DPABI) software (48, 49). Due to abnormal anatomical scans or visible artifacts in the images caused by excessive head movement, the functional data were deleted. Discarding each participant’s first ten volumes made the machine signal stable and participants were adapted to scanning noise. The remaining volumes were processed for slice timing correction. Then, the computer automatically rearranged images and assessed movement parameters for each participant. The head motion amplitude of each subject was evaluated by the frame-wise displacement (FD). In the present study, FD was set to 0.5 (mean FD ± twice of standard deviation) (50). Due to the large differences in shape and size of different individuals’ brains, it was difficult to directly make a comparison. Registration and standardization were carried out *via* mapping of different subjects’ brains into a space with the same shape and size so that to make the cerebral anatomical structures of subjects corresponded to each other. The linear trend of covariates was removed and corrected with Friston-24 (51). We regressed out several nuisance covariates, such as the global brain signal, the white matter signal, and the cerebrospinal fluid signal (50). DARTEL was used to standardize different brains and to increase the comparability of cerebral functional data among subjects (52). Finally, 6 mm full-width at half-maximum (FWHM) was used for smoothing to improve the reliability of data.

However, 7 first-episode drug-naïve patients with SZ and three HCs were excluded due to excessive head movement when MRI scans were performed.

### The Fractional Amplitude of Low-Frequency Fluctuations Analysis

Voxel-wise fALFF maps were scanned for each participant using the rs-fMRI machine. ALFF reflects the spontaneous activity of the local cerebral region. The time series of each voxel was first transformed into the frequency domain by the fast Fourier transform. The amplitudes were measured using fALFF within the slow-4 (range, 0.027–0.073 Hz) and slow-5 (range, 0.01–0.027 Hz) bands. FALFF was calculated by the ratio of the root mean square of the power spectrum of low-frequency (range, 0.01–0.08 Hz) to the root mean square of the power spectrum of the whole frequency (range, 0–0.25 Hz) (37, 40). The fALFF analysis was carried out to reduce the influences of low-frequency drift and high-frequency physiological noise by dividing the total value of full-band amplitude from 0.01 to 0.25 Hz (53).

### The Resting-State Functional Connectivity Analysis Based on Orbitofrontal Cortex Seeds

The rsFC was calculated with the Fisher’s transformation of the correlation coefficient using seed-to-voxel analysis (50). The rsFC analysis was performed on the abnormal region (the left OFC) with global voxels. Connectivity maps were obtained for the left OFC by correlating its average time series with every voxel in the brain.

### Statistical Analysis

Group-level analyses were carried out to examine cerebral regions with detectable fALFF abnormalities in SZ. The two-sample *t*-test was used to compare fALFF between SZ and HC groups. The fALFF maps were calculated using permutation-based non-parametric inference with 5,000 permutations, with consideration of age, gender, and educational level as covariates of no interest. The statistical threshold was set at voxel level (*p* < 0.001) or cluster level (*p* < 0.05), and the correction was conducted for multiple comparisons by the threshold-free cluster enhancement (TFCE) (54). The rsFC between the region of interest (ROI) and global brain voxels was corrected by the TFCE (with 5,000 permutations; voxel level, *p* < 0.001; cluster level, *p* < 0.05; with consideration of age, gender, and educational level as covariates of no interest) to avoid multiple comparison corrections. The significant fALFF decreased areas in the patients were used as inclusion masks in the following voxel-wise correction analyses, which were carried out to explore the correction between the fALFF map and the severity of symptoms (five components of PANSS). The statistical threshold was also set at voxel level (*p* < 0.001 or cluster level (*p* < 0.05) in the TFCE. Age, gender, educational level, and illness duration were entered into the model as covariates of no interest. Pearson correlation analysis was then used to assess the possible clinical relevance between values of rsFC and the severity of clinical symptoms. PANSS items were converted into five factors. Associations between rsFC and PANSS scores were carried out by Pearson’s correlations, applying Bonferroni correction for the number of correlations (5 subscale scores at *p* = 0.05/5 subscales = 0.01) (55). When other clinical symptoms are also related to rsFC, it is necessary to controlled significant correlations for the impact of other symptoms. If there was a significant correlation among PANSS excited scores, *post hoc* analysis was employed to explore the correlation among the 2 excited sub-item scores. Although PANSS excited scale contains four groups of clinical symptoms, we only used two dimensions (hostility and impulsivity) for correlation analysis. Associations between rsFC and PANSS excited subscale score were carried out by Pearson’s correlations, also applying Bonferroni correction for 2 comparisons (2 subscale scores at *p* = 0.05/2 = 0.025).

Age, gender, educational level, and illness duration were entered into the models as covariates of no interest. Statistical analysis was performed using SPSS 21.00 software (IBM, Armonk, NY, United States). The two-sample *t*-test and the Chi-square test were used for comparing differences in continuous variables and categorical variables between the two groups, respectively. *p* < 0.05 was considered statistically significant.

## Results

### Comparison of Demographic Data Between Schizophrenia and Healthy Control Groups

Demographic characteristics of 83 subjects belonging to SZ and HC groups were compared. The detailed demographic characteristics of subjects in the SZ and HC groups are presented in Table 1. There was no significant difference in gender (Chi-square test, χ*^2^* = 0.592, *p* = 0.441), age (two-sample *t*-test, *t* = −1.555, *p* = 0.08), and educational level (two-sample *t*-test, *t* = 1.569, *p* = 0.651) between the two groups.

**TABLE 1:** Demographic data and clinical information in patients with FES and health controls.

Variables	FES (*n* = 56)^a^	HCs (*n* = 27)^a^	t/χ ^2^	*p-*value
*P-value* Gender (Male/female)	38/18	16/11	0.592	0.441^b^
Age (years) Education (years) Illness duration (months)	23.64 ± 5.55 12.41 ± 3.10 9.79 ± 8.56	21.93 ± 3.23 13.67 ± 2.62 N/A	−1.555 1.569 N/A	0.08^c^ 0.651^c^ N/A
**PANSS**				
Total Positive factors Negative factors Disorganized factors Excited factors Depressed factors	107.93 ± 18.83 15.41 ± 3.69 24.64 ± 7.61 10.54 ± 3.29 10.09 ± 3.78 8.93 ± 3.78	N/A N/A N/A N/A N/A N/A	N/A N/A N/A N/A N/A N/A	N/A N/A N/A N/A N/A N/A

*Values are expressed as means ± standard deviations. There is no significant difference in gender, sex, and education (all p-values > 0.05).*

*FES, first-episode schizophrenia; HCs, healthy controls; PANSS, Positive and Negative Syndrome Scale; N/A, not applicable.*

*^a^Data from 83 subjects (56FES, 27HCs) are included due to the good quality of normalized images of fALFF.*

*^b^Two-tailed t-tests.*

*^c^Two-tailed chi-square tests.*

### Comparison of Fractional Amplitude of Low-Frequency Fluctuations Values

**FIGURE 1:**
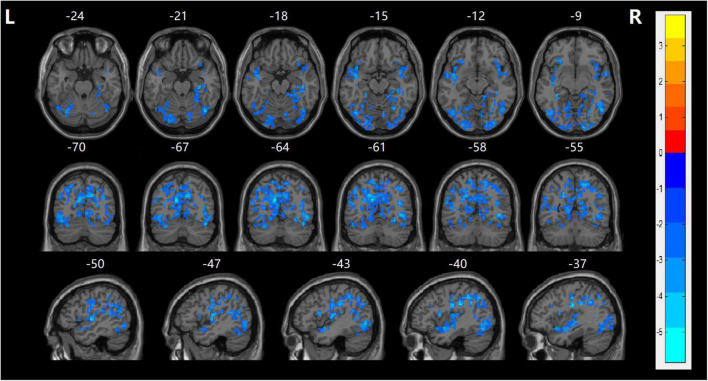
fALFF maps show differences between the naïve patients with FES and HCs (*p* < 0.05). The patients with FES showed significantly lower values in the bilateral precentral gyrus, bilateral postcentral gyrus, right lingual gyrus, bilateral temporal superior gyrus, left middle occipital gyrus, right calcarine, and left orbitofrontal cortex (LOFC) relative to both the HC group. Statistically significant differences in fALFF were defined as voxel-level, *p* < 0.001; cluster level, *p* < 0.05, TFCE corrected after correcting for age, gender, and education. T, statistical value of peak voxel showing fALFF values differences between the two groups. The cool color indicated that it decreased in the naïve patients with FES. Reversely, warm color indicated increased values in the naïve patients with FES.

In the current study, the fALFF values in several cerebral regions in the SZ group were lower than those in the HC group (TECE corrected, voxel-level, *p* < 0.001; cluster level, *p* < 0.05). Decreased fALFF values were found in the frontal and occipital lobes, including bilateral precentral gyrus, bilateral postcentral gyrus, right lingual gyrus, bilateral temporal superior gyrus, and left middle occipital gyrus, right calcarine, and left orbitofrontal cortex (LOFC) (Table 2 and Figure 1). There were no significant differences in fALFF values among the other cerebral regions.

**TABLE 2:** Brain regions with decreased fALFF values in patients with FES compared with healthy controls (SCZ < HC).

Region	Cluster (voxel)	MNI coordinates (mm)			*T*-value
		X	Y	Z	
Right precentral gyrus	331	60	−3	24	−4.686
Left precentral gyrus	268	−59	0	11	−3.506
Right postcentral gyrus	228	64	−12	22	−4.197
Left postcentral gyrus	227	−62	−19	23	−5.288
Right lingual gyrus	214	15	−50	6	−4.454
Left temporal superior gyrus	209	−53	2	−1	−4.000
Left middle occipital gyrus	185	−33	−91	−3	−3.921
Right calcarine	174	10	−89	9	−3.507
Left orbitofrontal cortex	152	−29	27	−10	−3.694
Right temporal superior gyrus	149	52	−16	7	−4.220

*Statistically significant differences in fALFF were defined as p < 0.05, TFCE corrected after correcting for age, gender, and education.*

*fALFF, fractional low-frequency amplitude; FES, first-episode schizophrenia; MNI, Montreal Neurological Institute; x, y, z, are the coordinates of the primary peak locations in the MNI space; T is the statistical value of the peak voxel showing the significant fALFF differences among all the groups.*

### Comparison of Resting-State Functional Connectivity Values

**FIGURE 2:**
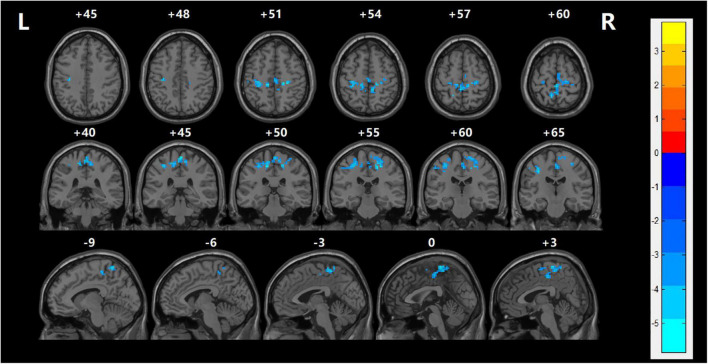
The resting-state functional connection was analyzed between the left orbitofrontal cortex and the global brain. With regards to the HC group, left OFC had lower rsFC values in several regions including the left postcentral gyrus, left precuneus, left superior temporal gyrus, and left precentral gyrus with FES (TECE corrected, voxel-level, *p* < 0.001; cluster level, *p* < 0.05). The cool color indicates that rsFC is decreased in the naïve patients with FES. Reversely, warm color indicates rsFC is higher than in the healthy control group.

Functional connectivity between the LOFC and each voxel of the global brain was performed to examine network abnormalities in the SZ group. After making correction for multiple comparisons, patients in the SZ group demonstrated significantly reduced rsFC values between the LOFC and several cerebral regions, which were mainly distributed in the bilateral postcentral gyrus, right middle frontal gyrus, left precentral gyrus, bilateral paracentral lobule, and right median cingulate compared with HC group (TECE corrected, voxel-level, *p* < 0.001; cluster level, *p* < 0.05) (Table 3 and Figure 2).

**TABLE 3:** Coordinates the peaks of the brain regions with decreased rsFC in naïve schizophrenic patients.

ROI	Region	Cluster (voxel)	MNI coordinates (mm)			*T*-value
			*X*	*Y*	*Z*	
LOFC	Left postcentral gyrus	53	−31	−33	52	−4.222
LOFC	Right middle frontal gyrus	53	27	−24	60	−3.756
LOFC	Left precentral gyrus	50	−21	−27	55	−3.882
LOFC	Right paracentral lobule	49	0	−34	69	−4.364
LOFC	Right postcentral gyrus	49	20	−25	66	−3.659
LOFC	Left paracentral lobule	38	−13	−29	51	−4.970
LOFC	Right median cingulate	18	18	−27	48	−5.194

*rsFC, resting-state functional connection; ROI, region-of-interest; LOFC; left orbitofrontal cortex; MNI, Montreal Neurological Institute; x, y, z, are the coordinates of the primary peak locations in the MNI space; T-value of the peak voxel showed different functional connectivity with the left frontal orbital gyrus seed in the patients compared to healthy controls (TECE corrected, voxel-level, p < 0.001; cluster level, p < 0.05).*

### Correlations Between the Fractional Amplitude of Low-Frequency Fluctuations Values and Positive and Negative Syndrome Scale Scores

To detect possible associations of spontaneous cerebral activity with psychiatric symptoms, the PANSS was used for the assessment of symptoms, which included symptoms in 5 dimensions (55). These symptoms were correlated with cerebral activation and behavioral activities in patients in the SZ group. It was attempted to elucidate the relationship between the fALFF values and PANSS scores (five dimensions of symptoms) using age, gender, educational level, and illness duration as covariates. However, no voxel has existed after TFCE correction (voxel-level, *p* < 0.001; cluster level, *p* < 0.05).

### Correlations Between the Resting-State Functional Connectivity Values and Positive and Negative Syndrome Scale Scores

**FIGURE 3:**
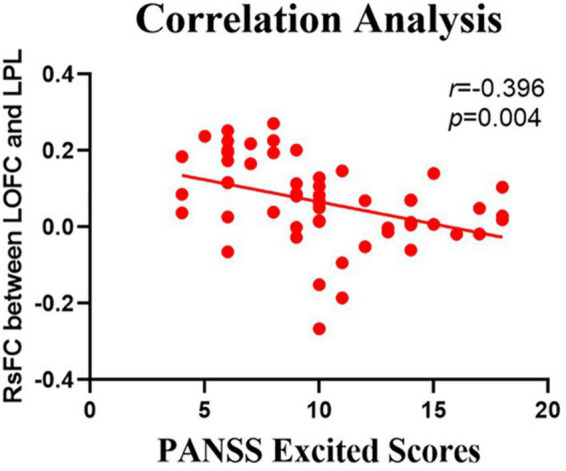
Negative correlation between the resting-state functional connectivity (rsFC) values and the severity of excited symptoms in the FES. The X-axis is the score of the excited factors of the PANSS scale, and the Y-axis shows the functional connectivity of the left orbitofrontal cortex (LOFC) and left paracentral lobule (LPL) (after correcting for positive symptoms, age, gender, education, and illness duration).

**FIGURE 4:**
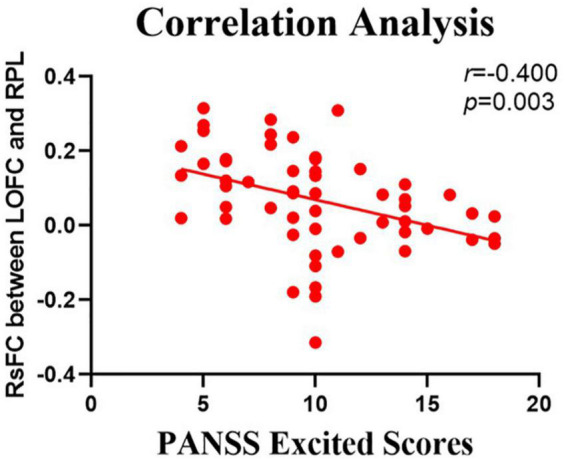
Negative correlation between the resting-state functional connection (rsFC) values and the severity of excited symptoms in the FES. The X-axis is the score of the excited factors of the PANSS scale, and the Y-axis shows the functional connectivity of the left orbitofrontal cortex (LOFC) and right paracentral lobule (RPL) (after correcting for positive symptoms, age, gender, education, and illness duration).

**FIGURE 5:**
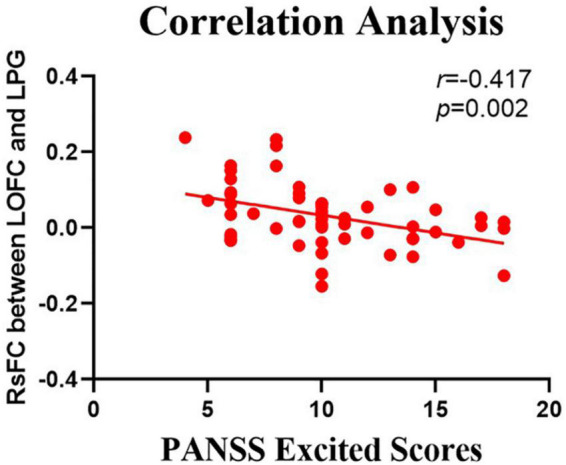
Negative correlation between the resting-state functional connectivity (rsFC) values and the severity of exited scores in the FES. The X-axis is the score of the excited factors of the PANSS scale, and the Y-axis shows the functional connectivity of the left orbitofrontal cortex (LOFC) and left postcentral gyrus (after correcting for positive symptoms, age, gender, education, and illness duration).

The relationship between the rsFC values and PANSS scores in patients in the SZ group was assessed (Tables 4, 5). There were several significantly negative correlations between the rsFC values and PANSS excited scores (five dimensions of symptoms) after Bonferroni correction (significant after Bonferroni correction for 5 correlations, all *p* < 0.05/5). After consideration of age, gender, educational level, and illness duration as covariates, these significantly negative correlations could be summarized as follows (Table 5): (i) the LOFC and the left paracentral lobule (*r* = −0.396, *p* = 0.004, Figure 3), (ii) the LOFC and the right paracentral lobule (*r* = −0.400, *p* = 0.003, Figure 4), (iii) the LOFC and the left postcentral gyrus (*r* = −0.417, *p* = 0.002, Figure 5), (iv) LOFC and the right postcentral gyrus (*r* = −0.354, *p* = 0.01). However, PNASS excited scores did not similarly exhibit a significantly negative correlation with the rsFC values between the LOFC and the right postcentral gyrus, when age, gender, educational level, and illness duration were not included as covariates (*r* = −0.326, *p* = 0.014, Table 4).

**TABLE 4:** Correlations between the rsFC values and PANSS excited scores in patients with FES.

PANSS	rsFC	*r*	*p*
Excited scores	LOFC-right median cingulate	−0.274	0.041*
Excited scores	LOFC-left paracentral lobule	−0.387	0.003**
Excited scores	LOFC-right paracentral lobule	−0.401	0.002**
Excited scores	LOFC-left precentral gyrus	−0.299	0.025*
Excited scores	LOFC-right precentral gyrus	−0.311	0.02*
Excited scores	LOFC-right postcentral gyrus	−0.326	0.014*
Excited scores	LOFC-left postcentral gyrus	−0.378	0.004**

*Results are significant at p < 0.05, **results are significant at p < 0.01. rsFC, resting-state functional connection; LOFC, left orbitofrontal cortex.

**TABLE 5:** Correlations between the rsFC values and PANSS excited scores in patients with FES (taking age, gender, education, and illness duration into covariates).

PANSS	rsFC	*r*	*p*
Excited scores	LOFC-right median cingulate	−0.308	0.026*
Excited scores	LOFC-left paracentral lobule	−0.396	0.004**
Excited scores	LOFC-right paracentral lobule	−0.400	0.003**
Excited scores	LOFC-left precentral gyrus	−0.330	0.017*
Excited scores	LOFC-right precentral gyrus	−0.275	0.048*
Excited scores	LOFC-right postcentral gyrus	−0.354	0.01**
Excited scores	LOFC-left postcentral gyrus	−0.417	0.002**

*Results are significant at p < 0.05, **results are significant at p < 0.01. rsFC, resting-state functional connection; LOFC, left orbitofrontal cortex.

The impact of symptoms other than excited ones on these correlation results was considered. There was a weak negative correlation (*r* = −0.282, *p* = 0.043, Supplementary Table 1) between positive symptoms and rsFC (left OFC—left postcentral gyrus). There was no significant correlation between rsFC and negative, cognitive as well as depressed symptoms (all *p* > 0.05). In order to control the impact of positive symptoms on correlation analysis, positive symptoms, age, gender, course of the disease, and years of education were taken as covariates, excited symptoms were still negatively correlated with rsFC. These negative correlations could be summarized as follows (Supplementary Table 2): (i) the LOFC and the left paracentral lobule (*r* = −0.327, *p* = 0.019), (ii) the LOFC and the right paracentral lobule (*r* = −0.343, *p* = 0.014), (iii) the LOFC and the left postcentral gyrus (*r* = −0.347, *p* = 0.013).

### Correlations Between the Resting-State Functional Connectivity Values and Positive and Negative Syndrome Scale Excited Subscale Scores

The relationship between the rsFC values and PANSS excited subscale scores in patients in the SZ group was assessed (Tables 6, 7). There were several significantly negative correlations between the rsFC values and hostility (two dimensions of symptoms) after Bonferroni correction (significant after Bonferroni correction for 2 correlations, all *p* < 0.05/2). Significantly negative correlations were found between the LOFC and the bilateral paracentral lobule, as well as between the rsFC value of the right median cingulate and hostility symptoms (*r* = −0.369, *p* = 0.007, *r* = −0.328, *p* = 0.018, *r* = −0.459, *p* = 0.001, gender, age, and illness duration were taken as covariates into account, Table 7). Impulsivity symptoms showed negative correlations with the rsFC value of the right paracentral lobule and left postcentral gyrus (*r* = −0.327, *p* = 0.018, *r* = −0.315, *p* = 0.023, gender, age, and illness duration were taken as covariates into account, Table 7).

**TABLE 6:** Correlations between the rsFC values and PANSS excited subscale scores in patients with FES.

Excited symptoms	rsFC	*r*	*p*
Hostility	LOFC-right median cingulate	−0.407	0.002**
Hostility	LOFC-left paracentral lobule	−0.316	0.017*
Hostility	LOFC-right paracentral lobule	−0.333	0.012**
Impulsivity	LOFC-right paracentral lobule	−0.332	0.012**

*Results are significant at p < 0.05, **results are significant at p < 0.025. rsFC, resting-state functional connection; LOFC, left orbitofrontal cortex.

**TABLE 7:** Correlations between the rsFC values and PANSS excited subscale scores in patients with FES (after correcting for age, gender, education, and illness duration).

Excited symptoms	rsFC	*r*	*P*
Hostility	LOFC-right median cingulate	−0.459	0.001**
Hostility	LOFC-left paracentral lobule	−0.328	0.018*
Hostility	LOFC-right paracentral lobule	−0.369	0.007**
Impulsivity	LOFC-right paracentral lobule	−0.327	0.018*
Impulsivity	LOFC-left postcentral gyrus	−0.315	0.023*

*Results are significant at p < 0.05, **results are significant at p < 0.012. rsFC, resting-state functional connection; LOFC, left orbitofrontal cortex.

There were several significantly negative correlations between the rsFC values and PANSS excited subscale scores (hostility and impulsivity) after consideration of positive symptoms, age, gender, educational level, and illness duration as covariates. These significantly negative correlations of hostility could be summarized as follows (Supplementary Table 3): (i) the LOFC and the right paracentral lobule (*r* = −0.332, *p* = 0.017), (ii) the LOFC and the right postcentral gyrus (*r* = −0.321, *p* = 0.022), (iii) the LOFC and the right median cingulate (*r* = −0.428, *p* = 0.002), (iv) the LOFC and the left precentral gyrus (*r* = −0.328, *p* = 0.019), (v) the LOFC and the right precentral gyrus (*r* = −0.318, *p* = 0.023). Impulsivity symptoms showed negative correlations with the rsFC value of the right paracentral lobule and left precentral gyrus (*r* = −0.335, *p* = 0.016, *r* = −0.337, *p* = 0.015, *p* < 0.025, Supplementary Table 3). However, hostility did not similarly exhibit a significantly negative correlation with the rsFC values, when positive symptoms, age, gender, educational level, and illness duration were not included as covariate. These not significant negative correlations were summarized as follows (Table 6): (i) the LOFC and the right postcentral gyrus, (ii) the LOFC and the right precentral gyrus, (iii) the LOFC and the left precentral gyrus. Impulsivity symptoms showed negative correlations with the rsFC value of the right paracentral lobule and left precentral gyrus (*r* = −0.335, *p* = 0.016, *r* = −0.337, *p* = 0.015, *p* < 0.025, Table 7).

## Discussion

We, in the present study, utilized rs-fMRI data, including fALFF and fALFF-based rsFC values to measure the resting-state of spontaneous cerebral functions in first-episode drug-naïve patients with SZ who did not experience antipsychotic medication. The results initially confirmed that compared with HCs, first-episode drug-naïve patients with SZ exhibited decreased fALFF values in the LOFC, bilateral precentral gyrus, and bilateral postcentral gyrus. In the second step, the LOFC was taken as a seed into account to explore rsFC values in first-episode drug-naïve patients with SZ. We found decreased rsFC values between the LOFC and frontal cortex, including bilateral postcentral gyrus, right middle frontal gyrus, bilateral paracentral lobules, the left precentral gyrus, and the right median cingulate. Finally, several correlation analyses were conducted to indicate whether the functional connectivity in the LOFC could be negatively correlated with excited symptoms, especially impulsive behaviors, and hostility. Considering the influences of potential confounding factors of the course of the disease and drugs, our results may provide a possible direction for exploring the pathogenesis of excited symptoms in first-episode drug-naïve patients with SZ.

FALFF or ALFF is an index that always measures the spontaneous activity of the cerebral regions, reflecting the levels of cerebral blood flow (CBF) and glucose metabolism. The decreased fALFF value in the LOFC in first-episode drug-naïve patients with SZ was previously reported repeatedly (23, 35). To our knowledge, OFC is important for the assessment of thoughts and intentions on social communication by information flow integration and feedback (3, 5, 56). The fALFF value reflects the speed or amount of information flow in OFC. Neuroimaging studies indicated that the density of the LOFC gray matter also decreased in first-episode drug-naïve patients with SZ (4, 26, 57). The loss of gray matter volume was reported to be associated with N-methyl-D-aspartic acid (NMDA) dysfunction and dopamine hyperfunction, which activated neurotoxic signal transduction pathways and induced neuronal, and synaptic necrosis (5). This triggered off the compressing or blocking of the transmitted information in varying degrees, explaining the functional deactivation state of OFC. The same decreased fALFF value in the LOFC was also found in patients with impulsive SZ (30). The impaired OFC decreased the inhibitory control mechanism through GABAergic neurons, and the change of OFC might lead to the delay of impulse inhibition (18). Hoptman et al. found the increased fALFF values of OFC in patients with chronic SZ (58), which was contrary to our results. Further study suggested that antipsychotics could be effective by partially restoring the function of the frontal cortex through synaptic plasticity (35, 59). The reason for the increase in fALFF value may be that the chronic patients received medication, and the function of OFC was partially recovered and played a super-compensatory role (58). These findings suggested that the anomalous OFC function in SZ patients may be related to the time of onset.

Numerous studies demonstrated that SZ is a functional connectivity disorder, and our research provided new evidence for this statement (59, 60). Anatomically, the paracentral lobule is the medial extension of the precentral and postcentral gyri (61). Precentral gyrus and postcentral gyrus, as well as paracentral lobule possess a consistency in segregating and conveying sensorimotor information, and they are the main components of the sensorimotor network (SMN) (56, 62). Recently, it was suggested that a sufficient information flow in SMN is essential to ensure unbiased emotional and behavioral responses (63). Several studies have reported the associations of impulsive violence to motor errors and the reduced speed of perceptual information processing in daily life (21).

The cingulate cortex is a structure implicated in behavioral adaptation and control. The median cingulate cortex is highly connected to areas involved in action control and decision making, such as OFC, dorsolateral prefrontal cortex, sensorimotor, and motor cortices. The median cingulate cortex has been emphasized to play a role in different facets of cognitive control, including response selection, attentional processing, conflict monitoring, and detecting errors (64). Our results also confirmed that the functional association between the LOFC and the right median cingulate is sparse in first-episode drug-naïve patients with SZ. It was demonstrated that the median cingulate cortex harbors a large number of short cortical contact fibers connected to the frontal cortex (65). The decreased connectivity indicated impaired sensorimotor integration and motor control in the early onset (66, 67).

To better evaluate the relationship between excited symptoms and cerebral functional conditions, we further proved that the functional connection between OFC and SMN including bilateral paracentral lobules, as well as median cingulate was negatively correlated with the scores of excited symptoms. The thickness of the gray matter cortex of OFC and paracentral lobule was smaller, which was related to its perceived function in SZ (68). Anatomical studies demonstrated defects in the cingulate cortex and OFC that were bound up with impulsive behaviors and hostility in psychiatric patients and HCs (27, 52, 69–71). Due to the role of the paracentral lobule in transmitting sensory and motor information to the SMN, an abnormal connectivity could lead to misperceptions and inappropriate behaviors. People could be fully mobilized flexibility through the OFC to adapt emotions to environmental changes, and functional connectivity might lead to disease-related painful experience and expressions of abnormal emotions or behaviors. We analyzed this correlation and clarified the changes in the brain network in the early stage of SZ, and clinical excited symptoms of SZ could be attributed to abnormal OFC-SMN circuits (56). Reduced functional connectivity between OFC and MCC could lead to an attenuated ability to monitor conflicts and detect errors (64). Functional deactivation of relevant cerebral regions, reduced information flow, difficulty in synchronizing activity between cerebral regions, decreased connectivity, and other dysfunctions could occur continuously in first-episode drug-naïve patients with SZ. All these constitute the neuropathological basis of excited symptoms. Psychotic symptoms including excited symptoms could be effectively relieved by adherence to antipsychotic medication during the acute phase of the disease. As the target of 5-OH and DA, OFC had been shown to partially restore its function (34, 35). However, long-term antipsychotic treatment had not achieved sustained results. The future research will concentrate on performing effective and accurate treatments.

In the subsequent analysis, we also observed a significantly negative correlation between connectivity strength in OFC and hostility symptoms in first-episode drug-naïve patients with SZ (*P* < 0.025). Similarly, the correlation between impulsivity and functional connection decreased, while the significance was not comparable to hostility. The possible reason is that abnormal connectivity of the OFC circuit enhanced the reactivity of negative stimuli (72), and was further considered to increase the tendency of personal hostility and impulsivity (73). Patients in our study had the first onset of SZ. The spontaneous activity and functional connection of OFC decreased, resulting in mild agitation symptoms, such as hostility. When the course of the disease continued to progress, they further showed impulsive behaviors. Based on the negative correlation, the level of functional connection between OFC and SMN deviated from the normal, representing the severity of excited symptoms. We could make full use of this result to further predict the risk of impulsive behaviors in patients with SZ. Such accurate prediction and control measures may enable us to reduce social and public hazards.

## Limitations

There are several limitations in the present study. First, the study used a cross-sectional design and could not forecast how cerebral spontaneous activity may change during the clinical course of SZ. Further studies are therefore needed to fully understand the implications of our findings. Second, the sample size of our research was small, although it was comparable to most fMRI studies on first-episode drug-naïve patients with SZ, and it is essential to enrich the theoretical basis. Third, we clarified the correlation between LOFC-SMN connection and excitatory symptoms. However, it remained elusive whether the pathophysiology of excitatory symptoms originates from LOFC, SMN, or LOFC-SMN. Last but not least, our research concentrated on excited symptoms, while the severity of the symptoms varied among subjects, and the monotonic function of the measurement tool was noteworthy.

## Conclusion

In summary, first-episode drug-naïve patients with SZ were found to be associated with the decreased spontaneous cerebral activity in the LOFC. The rsFC values, in association with the LOFC, were significantly correlated with excited symptoms. More importantly, we determined that these coupling strengths could early predict the violent behaviors exhibited by these patients. Our findings might provide some perspectives for the study of hostility and impulsivity in first-episode drug-naïve patients with SZ.

## Data Availability Statement

The raw data supporting the conclusions of this article will be made available by the authors, without undue reservation.

## Ethics Statement

The studies involving human participants were reviewed and approved by the Medical Research Ethics Committee of Nanjing Brain Hospital. The patients/participants provided their written informed consent to participate in this study.

## Author Contributions

YS and JY designed the study. JY and YLv conducted the literature searches and analysis. CC and JL performed the statistical analysis. XXZ and XYZ managed the assessment of the risk of bias. CC wrote the manuscript. JL contributed to the final review of the manuscript. YLi provided a lot of useful feedback and helped modify the wording and syntax of the article during the revision of the manuscript. All authors contributed to the article and approved the submitted version.

## Conflict of Interest

The authors declare that the research was conducted in the absence of any commercial or financial relationships that could be construed as a potential conflict of interest.

## Publisher’s Note

All claims expressed in this article are solely those of the authors and do not necessarily represent those of their affiliated organizations, or those of the publisher, the editors and the reviewers. Any product that may be evaluated in this article, or claim that may be made by its manufacturer, is not guaranteed or endorsed by the publisher.
