# Supplementary material for: Aberrant Functional Connectivity of the Orbitofrontal Cortex Is Associated With Excited Symptoms in First-Episode Drug-Naïve Patients With Schizophrenia
Source: Front Psychiatry. 2022 Jul 28;13:922272. doi: 10.3389/fpsyt.2022.922272 (PMC9366470; doi:10.3389/fpsyt.2022.922272)
Supplement: Supplementary file 1 [file Table_1.DOCX]

**Supplementary materials**

**Supplementary Table 1** Correlations between the rsFC values and PANSS scores in patients with FES.

| PANSS | rsFC | r | p |
| --- | --- | --- | --- |
| Positive scores | LOFC- Left postcentral gyrus | -0.282 | 0.043^*^ |
| Negative scores | LOFC-Left Precentral Gyrus | -0.136 | 0.336 |
| Cognitive scores | LOFC- Left postcentral gyrus | -0.181 | 0.200 |
| depressed scores | LOFC- Left postcentral gyrus | -0.189 | 0.159 |

^*^Results are significant at p < 0.05, rsFC, resting-state functional connection. LOFC, left orbitofrontal cortex.

**Supplementary Table 2** Correlations between the rsFC values and PANSS excited scores in patients with FES (taking positive symptoms, age, gender, education, and illness duration into covariates).

| PANSS | rsFC | r | p |
| --- | --- | --- | --- |
| Excited scores | LOFC-Right Median Cingulate | -0.241 | 0.088 |
| Excited scores | LOFC-Left Paracentral Lobule | -0.327 | 0.019^*^ |
| Excited scores | LOFC-Right Paracentral Lobule | -0.343 | 0.014^*^ |
| Excited scores | LOFC-Left Precentral Gyrus | -0.243 | 0.086 |
| Excited scores | LOFC-Right Precentral Gyrus | -0.289 | 0.04^*^ |
| Excited scores | LOFC-Right Postcentral Gyrus | -0.317 | 0.023^*^ |
| Excited scores | LOFC-Left Postcentral Gyrus | -0.347 | 0.013^*^ |

^*^Results are significant at p < 0.05, rsFC, resting-state functional connection. LOFC, left orbitofrontal cortex.

**Supplementary Table 3** Correlations between the rsFC values and PANSS excited subscale scores in patients with FES. (taking positive symptoms, age, gender, education, and illness duration into covariates).

| Excited symptoms | rsFC | r | p |
| --- | --- | --- | --- |
| Hostility | LOFC-Right Median Cingulate | -0.428 | 0.002^**^ |
| Hostility | LOFC-Left Paracentral Lobule | -0.284 | 0.043^*^ |
| Hostility | LOFC-Right Paracentral Lobule | -0.332 | 0.017^**^ |
| Hostility | LOFC-Left Precentral Gyrus | -0.328 | 0.019^**^ |
| Hostility | LOFC-Right Precentral Gyrus | -0.318 | 0.023^**^ |
| Hostility | LOFC-Right Postcentral Gyrus | -0.321 | 0.022^**^ |
| Impulsivity | LOFC-Right Middle Frontal Gyrus | -0.335 | 0.016^**^ |
| Impulsivity | LOFC-Left Precentral Gyrus | -0.337 | 0.015^**^ |

^*^Results are significant at p < 0.05, ^**^ results are significant at p < 0.025. rsFC, resting-state functional connection. LOFC, left orbitofrontal cortex.
